# Knowledge translation gaps that need to be bridged to enhance life for people with spinal cord injury

**DOI:** 10.1038/s41394-024-00634-5

**Published:** 2024-04-23

**Authors:** J. Melin, E. Axwalter, G. Åhrén, Å. Lundgren Nilsson, K. S. Sunnerhagen, J. Wangdell

**Affiliations:** 1 The Gothenburg Competence Centre for Spinal Cord Injury, University of Gothenburg and Sahlgrenska University Hospital, Gothenburg, Sweden; 2 RISE Research Institutes of Sweden, Division Safety and Transport, Department Measurement Science and Technology, Gothenburg, Sweden; 3 Department of Clinical Neuroscience, Sahlgrenska Academy, Institute of Neuroscience and Physiology, University of Gothenburg, Gothenburg, Sweden; 4 Department of Neurocare, Sahlgrenska University Hospital, Gothenburg, Sweden; 5 Department of Hand Surgery, Institute of Clinical Sciences, University of Gothenburg, Gothenburg, Sweden; 6 Centre for Advanced Reconstruction of Extremities, Sahlgrenska University Hospital/Mölndal, Gothenburg, Sweden

**Keywords:** Patient education, Rehabilitation

There is a ‘need to know’ for people who have acquired a spinal cord injury (SCI) [1] and a ‘lack of knowledge’ is considered to be a barrier to coping with SCI [2]. In-patient SCI care and rehabilitation have emphasized that information should be provided in the right amounts, at the right place and at the right time [3]. Such needs and timing probably go beyond initial care and rehabilitation. Likewise, there is also, reasonably a need for knowledge for other groups of people meeting people with SCI [4, 5], such as relatives, health professionals and personal care assistants^1^ as well as authorities, decision-makers, employers, architects and city planners etc. Our recent work [7], shows that more evidence is needed regarding the provision of specialist expertise in SCI by care services and to what extent they are therefore able to respond to the needs of people living with SCI.

A Swedish need assessment project, including a priority setting partnership (PSP^2^) according to James Lind Alliance [8], has been implemented nationwide 2021–22 to address the needs which are most important for people with SCI to enhance their lives. The Swedish need assessment project comprises three parts: (a) needs to be met; (b) questions to be answered; (c) knowledge required. In part (b), which is the PSP we have identified research priorities [7] (e.g. knowledge gaps); while in part (c), presented here, we report results from an online survey addressing knowledge translation gaps in order to shed light on the knowledge that is available yet which does not reach out.

An online survey was released in November–December 2021 with people with SCI, relatives of people with SCI and health professionals and care assistants working with people with SCI. This survey included an open-ended question concerning knowledge translation gaps – what kind of knowledge is lacking and who needs the knowledge. In total, 242 persons responded the survey. Table 1 provides personal characteristics. Each respondent could state one to five knowledge needs, resulting in 480 inputs (statements with more than one need were treated separately in the total number of inputs). However, 84 statements were removed due to beyond the scope of the study (e.g., addressing general needs not related to needs for knowledge). The statements where further complemented with 64 statements from the PSP question [7] (where these were asked for issues which were difficult to find answers to). Thus, this report is based on a total of 459 statements included in this study.

**Table 1:** Personal characteristics of respondents participating in the survey.

	People living with SCI	Relatives	Health professionals	Personal care assistants	Total
	*n* = 128 (53)	*n* = 34 (1)	*n* = 78 (32)	*n* = 3 (1)	*n* = 243
Statement	217 (47)	71 (15)	164 (35)	7 (2)	n = 459
Gender					
Male	70 (55)	6 (18)	15 (19)	0	91 (38)
Female	58 (45)	28 (82)	62 (81)	3 (100)	151 (62)
Missing	—	—	1	—	1
Age, years					
Mean (SD)	54 (11)	54 (12)	44(12)	36 (8)	51 (13)
Median (range)	55 (28–78)	54 (28–72)	45.5 (22–68)	33 (30–45)	51 (22–78)
<30	2 (2)	2 (1)	13 (18)	1 (33)	18 (8)
31–45	25 (20)	5 (17)	24 (32)	2 (66)	57 (24)
46–60	62 (50)	15 (50)	30 (41)	—	107 (45)
60–75	34 (27)	8 (27)	7 (9)	—	49 (21)
>75	2 (2)	—	—	—	—
Missing	3	4	—	—	7
Municipality group					
Large cities and nearby municipalities	54 (46)	16 (48)	28 (39)	1 (33)	99 (44)
Medium-sized towns and nearby municipalities	46 (39)	13 (39)	26 (36)	1 (33)	86 (38)
Small towns/urban areas and rural municipalities	18 (15)	4 (12)	18 (9)	1 (33)	41 (18)
*Missing*	10	1	6	—	16
Time post injury (TPI)					
Mean (SD)	19 (15)	—	—	—	—
Median (range)	16 (0–55)	—	—	—	—
1–5	26 (21)	—	—	—	—
6–10	17 (14)	—	—	—	—
11–15	18 (15)	—	—	—	—
16–20	12 (10)	—	—	—	—
21–25	5 (4)	—	—	—	—
26–30	8 (7)	—	—	—	—
>30	37 (30)	—	—	—	—
Missing	5	—	—	—	—
Cause of injury					
Traumatic	97 (80)	—	—	—	—
Non-traumatic	24 (20)	—	—	—	—
Missing	7	—	—	—	—
Type of injury					
Complete	55 (45)	—	—	—	—
Incomplete	67 (55)	—	—	—	—
*Missing*	6	—	—	—	—
Tetraplegia	61 (49)	—	—	—	—
Paraplegia	63 (51)	—	—	—	—
Missing	4	—	—	—	—
Relative					
Partner	—	18 (53)	—	—	—
Parent	—	12 (35)	—	—	—
Sibling	—	2 (6)	—	—	—
Child	—	1 (3)	—	—	—
Friend	—	1 (3)	—	—	—
Missing	—	—	—	—	—
Profession					
Occupational therapist	—	—	12 (16)	—	—
Physiotherapist	—	—	13 (18)	—	—
Social worker	—	—	2 (3)	—	—
Physician	—	—	9 (12)	—	—
Psychologist	—	—	1 (1)	—	—
Rehab assistant	—	—	2 (3)	—	—
Nurse	—	—	14 (19)	—	—
Assistant nurse	—	—	21 (32)	—	—
Missing	—	—	4	—	—

## A comprehensive framework for functioning, disability and health

The International Classification of Functioning, Disability and Health (ICF) is a comprehensive framework which classifies and describes functioning, disability and health in people with all kinds of diseases or conditions [9]. In addition to being a day-to-day tool and a research framework, the ICF can also guide the analysis of needs to develop plans and actives where specific effort is critical to ensure the provision of a health care system and a society supporting health [10]. Thus, the ICF framework was considered appropriate to shed light on the knowledge that is available but which is not reaching out.

Due to the comprehensive nature of ICF to fit all kinds of diseases or conditions, so-called ICF core sets have been recommended for specific diseases and conditions. For people with SCI, there are two key core sets; one for SCI in an early post-acute context [11], and one for SCI in a long-term context [12]. These were used in a directed content analysis [13] of the statements to ascertain what kind of knowledge is needed for an enhanced life for people with SCI. This was complemented by a gap analysis if the ICF core sets did not cover the statements relating to the knowledge translation gaps. In addition, this was further complemented with a manifest and conventional content analysis [13] to ascertain who needs the knowledge.

## What kind of knowledge is needed?

**Fig. 1:**
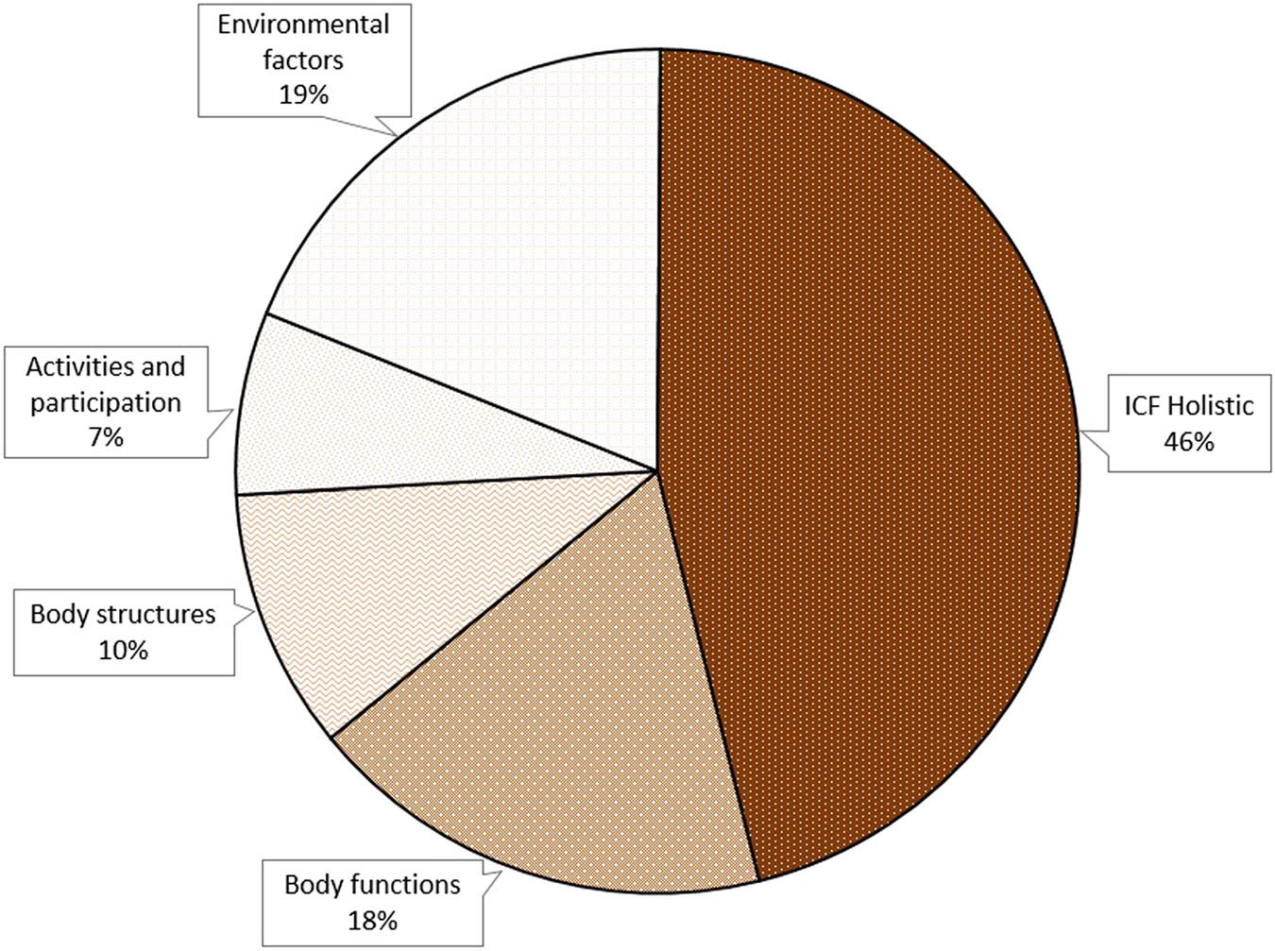
Pie chart showing distributions for those ICF components in which the knowledge is needed.

In total, 46% of the knowledge translation gaps could not be classified according to specific ICF components (Fig. 1). This could be interpreted as a need for knowledge relating to the holistic and comprehensive aspect of living with a SCI rather than the specific components. Of the knowledge translation gaps classified according to ICF, all components were represented expect for *Personal factors* (Fig. 1, Table 2).

**Table 2:** Knowledge translation gaps within each ICF component presented as numbers and percentages.

Component	Category	Secondary level code	n (% within component)
Body functions	b1 Mental functions	b122 Global psychosocial functions	2 (2)
		b130 Energy and drive functions	1 (1)
		b180 Experience of self and time functions	3 (4)
	b2 Sensory functions and pain	b280 Sensation of pain	18 (21)
	b5 Functions of the digestive, metabolic and endocrine systems	b525 Defecation functions	9 (11)
		b540 General metabolic functions	4 (5)
	b6 Genitourinary and reproductive functions	b620 Urination functions	12 (14)
		b650 Menstruation functions	1 (1)
		b660 Procreation functions	1 (1)
	b7 Neuromusculoskeletal and movement-related functions	b799 Neuromusculoskeletal and movement-related functions, unspecified	3 (4)
		*Other*	30 (36)
Body structures	s1 Structures of the nervous system	s120 Spinal cord and related structures	4 (9)
		s150 Structure of parasympathetic nervous system	11 (25)
		s198 Structure of nervous system, other specified	10 (23)
	s7 Structures related to movement	s770 Additional musculoskeletal structures related to movement	2 (5)
	s8 Skin and related structures	s810 Structure of areas of skin	16 (36)
		*Other*	1 (2)
Activities and participation	d2 General tasks and demands	d230Carrying out daily routine	4 (12)
	d4 Mobility	d460 Moving around within the home	1 (3)
	d5 Self care	d530 Toileting	1 (3)
		d540 Dressing	1 (3)
		d570 Looking after one’s health	3 (9)
	d7 Interpersonal interactions and relationships	d760 Family relationships	6 (18)
		d770 Intimate relationships	4 (12)
		d779 Particular interpersonal relationships, other specified and unspecified	2 (6)
	d8 Major life areas	d845 Acquiring, keeping and terminating a job	6 (18)
		d879 Economic life, other specified and unspecified	3 (9)
	d9 Community, social and civic life	d920 Recreation and leisure	1 (3)
		*Other*	1 (3)
Environmental factors	e1 Products and technology	e140 Products and technology for culture, recreation and sport	6 (7)
		e140 Products and technology for culture, recreation and sport	2 (2)
		e150 Design, construction and building products and technology of buildings for public use	13 (15)
		e199 Products and technology, unspecified	8 (9)
	e2 Natural environment and human-made changes to environment	e210 Physical geography	1 (1)
	e4 Attitudes	e430 Individual attitudes of people in positions of authority	1 (1)
		e440 Individual attitudes of personal care providers and personal assistants	1 (1)
		e445 Individual attitudes of strangers	5 (5)
		e450 Individual attitudes of health professionals	1 (1)
		e499 Attitudes, unspecified	1 (1)
	e5 Services, systems and policies	e525 Housing services, systems and policies	3 (3)
		e570 Social security services, systems and policies	25 (29)
		e575 General social support services, systems and policies	6 (7)
		e580 Health services, systems and policies	13 (15)

### A holistic view of the complexities of living with SCI is needed

The results of the survey clearly highlight a need to translate the holistic aspects of the complexities relating to living with SCI. Knowledge on specific aspects of SCI might be available, but the knowledge on how these pieces interact and add up might not be presented as often as is wished. Thus, the complexities need to be understood and described to enhance living with SCI. People living with SCI have a key function in this respect—to share their perspective with others living with SCI via peer support [14, 15], and through an involvement in setting the research agenda and establishing innovation and knowledge translation activities. It is important that this kind of peer involvement and work should not rely on individuals, rather on structured organizational and possibly institutional initiatives. In this way it is possible to ensure a high quality of the knowledge made accessible to any other individuals in need of peer support and involvement.

### Gaps in the ICF core sets for SCI

In this work, we identified aspects of knowledge translation gaps not covered in the ICF core sets for SCI [11, 12]. Some were non-specific possibly resulting from the particular nature of our survey. We found it however somewhat surprising that the section, *b180 Experience of self and time functions*, was not part of the ICF core sets for SCI [11, 12]. This is a common issue in the psychosocial rehabilitation after SCI [16–18]. In addition, our results indicate the ICF core sets do not cover important issues such as autonomic dysreflexia and incomplete SCI.

## Who should have what knowledge?

In less than one-third of the knowledge translation gaps there was no indication as to who needed the knowledge. In such cases, this was usually related to *Environmental factors* (32%, *n* = 42) and less commonly to *Body structures* (11%, *n* = 15). Among the knowledge translation gaps questions concerning who should have access to the knowledge, 39% suggested health professionals, followed by authorities and people living with SCI (Table 3). More specifically, the answers also indicated that the health care professionals beyond the SCI units (such as primary care, emergency departments and municipality care) were in the greatest need of knowledge.

**Table 3:** Who needs the knowledge translation presented as numbers and percentages.

Who	n (%)
People living with SCI	32 (10)
Health professionals	
Not SCI units	77 (24)
Physiotherapists and occupational therapists	11 (3)
Physicians	5 (2)
*Not specified*	34 (10)
Personal care assistants	13 (4)
Family members	20 (6)
Employers	13 (4)
Authorities	89 (27)
Others	33 (10)

### Reaching out to health care professionals beyond the SCI units

To facilitate knowledge of SCI research to be translated into clinical practice there are initiatives in place such as the Spinal Cord Injury Rehabilitation Evidence (SCIRE) Project [19, 20], which offers written information via their webpage relating to the available evidence and which assists with translating existing evidence into the clinical setting. Although this might not reach out to health professionals beyond the SCI units, further support is warranted in order to enhance life for people living with SCI. Thus, the health care professionals in the SCI units have a responsibility to share this complex knowledge with their colleagues working in other parts of the chain of care.

### Authorities and decision-makers need to understand the implications of an SCI in everyday life

In general, there were no major differences in terms of who should have access to the knowledge based on the answers from the respondents. However, one quarter of the responses from the people living with SCI stated that authorities (from relatives 15% and health professionals 26%) needed more knowledge. Thus, in this paper we also see a need for a different kind of knowledge translation than that typically provided to clinicians. Specifically, authorities and decision-makers need a better general understanding of the implications of SCI in everyday life and specific knowledge about each person with SCI to ensure he or she is given the right support. In turn, we believe that this should enable a better community integration and less social isolation as well as avoiding secondary health complications.

## When is the knowledge needed?

Our survey did not specify when the knowledge is needed, e.g., in the acute phase, in the rehabilitation phase or at any time later in life. Given the fact that the mean time post injury was 19 years, many knowledge translation gaps could also occur after the time spent at the SCI units. This speculation is also supported by the fact that both health care professionals beyond SCI units and authorities were considered to be persons and entities in need of more knowledge. Persons living with SCI often encounter these target groups post-discharge. On the other hand, it could also be the case that aspects of a non-medical nature such as personal care assistance, housing services, social security service and social support service etc. are not prioritised adequately in the initial phase. In Sweden, there is the challenge concerning the fact that this kind of adaptation and support is often regulated at a municipality level. Hospitals, on the other hand are regional entities which may confound the process of keeping up-to-date with the various different municipal regulations which are constantly changing and which are affecting their patients.

## A plea for more knowledge about living with SCI

This report confirms the well-known challenges associated with translating research evidence into practice. It makes the case for more knowledge about living with SCI. Specifically, a more holistic view of SCI is especially needed not only for people living with and affected by SCI and the health professionals and other staff at SCI units, but also to health professionals beyond just the SCI inpatient care and rehabilitation units (e.g., practitioners in primary care, professionals at municipality care and emergency departments) as well as decision-makers and politicians.

We would encourage research groups and other organizations to address methods for translating research evidence into practice and to consider the results of this report which indicate that the available knowledge is not reaching out to the right people. In forthcoming work, the knowledge translation gaps presented here will form a critical output from the planned Swedish need assessment project in order to enhance life for people living with SCI. As such, the results will be used to develop new educational and knowledge translation activities at the Gothenburg Competence Center for Spinal Cord Injury [21].

## Data Availability

The data in this study are available at OSF: MJA, EÅ, GS, KSN, ÅLW, J. (2023, January 26). Need assessment to enhance life for people living with SCI in Sweden – Needs, research priorities and knowledge transfer. 10.17605/OSF.IO/73APW.

## Footnotes

1 Under Swedish law [6], personal care assistance can be provided for people who, owing to serious and long-lasting functional impairments need help with meeting their own basic needs.

2 A Priority Setting Partnership (PSP) is a methodology developed by the James Lind Alliance (JLA), a non-profit making initiative, that brings patients, carers and clinicians together. The PSP comprising two main phases; an identification of uncertainties and a prioritization process.
